# Comparison of the Impact of COVID-19 on Veterans Affairs and Non-federal Hospitals: a Survey of Infection Prevention Specialists

**DOI:** 10.1007/s11606-022-07961-z

**Published:** 2022-11-30

**Authors:** Richard J. Schildhouse, Ashwin Gupta, M. Todd Greene, Karen E. Fowler, David Ratz, Mark S. Hausman, Sanjay Saint

**Affiliations:** 1 VA Ann Arbor Healthcare System, Ann Arbor, MI USA; 2 Department of Internal Medicine, University of Michigan Medical School, Ann Arbor, MI USA; 3 VA/UM Patient Safety Enhancement Program, Ann Arbor, MI USA; 4 Department of Anesthesiology, University of Michigan Medical School, Ann Arbor, MI USA

**Keywords:** COVID-19, health systems, health policy, system shock, personal protective equipment

## Abstract

**Background:**

As the COVID-19 pandemic evolves, it is critical to understand characteristics that have allowed US healthcare systems, including the Veterans Affairs (VA) and non-federal hospitals, to mount an effective response in the setting of limited resources and unpredictable clinical demands generated by this system shock.

**Objective:**

To compare the impact of and response to resource shortages to both VA and non-federal healthcare systems during the COVID-19 pandemic.

**Design:**

Cross-sectional national survey administered April 2021 through May 2022.

**Participants:**

Lead infection preventionists from VA and non-federal hospitals across the US.

**Main Measures:**

Surveys collected hospital demographic factors along with 11 questions aimed at assessing the effectiveness of the hospital’s COVID response.

**Key Results:**

The response rate was 56% (71/127) from VA and 47% (415/881) from non-federal hospitals. Compared to VA hospitals, non-federal hospitals had a larger average number of acute care (214 vs. 103 beds, *p*<.001) and intensive care unit (24 vs. 16, *p*<.001) beds. VA hospitals were more likely to report no shortages of personal protective equipment or medical supplies during the pandemic (17% vs. 9%, *p*=.03) and more frequently opened new units to care specifically for COVID patients (71% vs. 49%, *p*<.001) compared with non-federal hospitals. Non-federal hospitals more frequently experienced increased loss of staff due to resignations (76% vs. 53%, *p*=.001) and financial hardships stemming from the pandemic (58% vs. 7%, *p*<0.001).

**Conclusions:**

In our survey-based national study**,** lead infection preventionists noted several distinct advantages in VA versus non-federal hospitals in their ability to expand bed capacity, retain staff, mitigate supply shortages, and avoid financial hardship. While these benefits appear to be inherent to the VA’s structure, non-federal hospitals can adapt their infrastructure to better weather future system shocks.

## INTRODUCTION

In healthcare, a system shock has been defined as an organization-wide event that detracts from day-to-day operations.^1^ The COVID-19 pandemic has represented a major system shock to healthcare systems across the United States (US) in numerous ways such as limited staffing,^2,3^ lower revenue secondary to paused routine care, and ongoing medical supply chain disturbances.^4^ While resource constraints have been ubiquitous throughout the pandemic, neither the challenges experienced nor their impact on healthcare delivery has been distributed uniformly across healthcare delivery system types (e.g., federal and non-federal, urban and rural, academic and non-academic). For example, the Department of Veterans Affairs (VA)—the largest integrated healthcare system in the US consisting of more than 120 acute care hospitals—was called upon to provide support not only to Veterans, but also to overwhelmed communities as part of its “Fourth Mission.”^5^ Specifically, the VA’s Fourth Mission entailed opening its hospitals to non-Veteran patient admissions and transfers from community hospitals,^6^ along with providing personal protective equipment and personnel support to community, state, and federal healthcare facilities.

Infection prevention programs have proven to be both a critical component of healthcare systems’ COVID-19 response and have represented an area systemically understaffed nationally prior to the pandemic.^7^ The presence of a robust infection prevention program is doubly relevant given both the intensive operational management required to provide safe COVID-related care, and the finding that those affected with COVID-19 infection who require hospitalization are at substantially higher risk of acquiring healthcare-associated infections.^8^ For example, healthcare-associated infection rates for catheter-related bloodstream infections, catheter-associated urinary tract infections, and methicillin-resistant *Staphylococcus aureus* bloodstream infections rose 60%, 43%, and 44% above predicted levels throughout the pandemic, respectively, and correlated with hospital COVID-19 burden.^9^ As a result, infection preventionists have important insights into the response of hospitals to the COVID-19 pandemic.

While most hospitals have been affected by the COVID-19 pandemic, the extent to which organizational structure (e.g., the VA’s population health-based vs non-federal fee-for-service models) contributed to response effectiveness remains unknown. To assess differences in COVID-19 pandemic response between these two organizational structures, we surveyed infection preventionists from both VA and non-federal hospitals in the US.

## METHODS

### Study Design, Survey Instrument, and Data Collection

Questions about individual hospital’s COVID-19 pandemic response were added to an existing survey tool that has been administered previously to assess infection prevention practices within US hospitals.^10^ The survey instrument, administered every 4 years since 2005 and developed by Krein and colleagues,^11–14^ has undergone several revisions to accommodate changes in evidence and emerging areas of interest.^10,15,16^ Following each iteration, a national, stratified random sample of non-federal, general medical and surgical hospitals with an intensive care unit and all VA hospitals with general medical beds were surveyed. Before finalizing the sample, an internet search was conducted to confirm each facility’s operating status—closed facilities or those without general medical beds were removed from the mailing list. For the current survey period, surveys were sent to a total of 127 VA hospitals and to a random sample of 883 non-federal community hospitals. Of note, non-federal hospitals were eligible regardless of their reimbursement model or affiliation with a larger healthcare system. Participation by one site within a healthcare system did not preclude participation by other hospitals within the same network. Prior to survey distribution, a general informational email was distributed to all infection preventionists affiliated with the selected VA hospitals and by traditional mail addressed to the “Infection Control Coordinator” at each non-federal hospital notifying them of the forthcoming survey. The initial survey was sent in mid-April 2021 and a reminder was distributed via the previously used mechanism 2 weeks after initial survey distribution, requesting that the hospital infection preventionist (or supervising infection preventionist if there was more than one on staff) complete the survey. The survey could be completed on paper or via an electronic link to complete on a REDCap electronic survey platform.^17,18^ Each survey was labeled with a unique study number that provided hospital identification; however, the individual survey responders were anonymous. Additional reminder surveys were sent to non-responding hospitals approximately 1, 2, and 3 months following the initial survey distribution. The majority of surveys were returned by December 2021, with just three returned in early 2022. Two of the non-federal survey mailings were returned indicating the hospitals had closed and were therefore excluded from this analysis, leaving a total eligible sample of 881 non-federal hospitals. Appropriate institutional review board approval or exemption was obtained from both the University of Michigan and the VA Ann Arbor Healthcare System.

### Study Measures

The survey assessed general hospital characteristics, including the number of acute care beds, number of intensive care unit beds, affiliation with a medical school, the presence of a hospital epidemiologist, infection control program characteristics, and leadership support for infection control programs. The survey also assessed how hospitals have responded to COVID-19, the institutional challenges that the pandemic has imposed, sources of information used to guide COVID-19 responses, and infection preventionist’s perceptions surrounding COVID-19 vaccine and work environment safety during the pandemic. The survey assessed whether hospitals experienced “any shortages” of various medical supplies. The following survey questions, “In your opinion, how effective has your hospital’s pandemic response plan been in addressing COVID-19?” (Minimally effective, Moderately effective, Very effective, Extremely effective) and “Has your hospital experienced financial hardship resulting from the COVID-19 pandemic?” (No financial hardship, Mild financial hardship, Moderate financial hardship, Extreme financial hardship) were dichotomized during analyses to represent “very to extremely effective COVID response” and “moderate to extreme financial hardship.” For the COVID-19 questions, respondents were asked about their hospital’s experiences during the pandemic at any point from March 2020 until the present.

### Statistical Analysis

Descriptive statistics were calculated for hospital characteristics and specific COVID-19-related factors. Comparisons between VA and non-federal hospitals were made using *t*-tests for continuous variables and chi-square tests for categorical variables. All analyses were performed using SAS version 9.4 software (SAS Institute, Cary, NC) and *p*<.05 was considered statistically significant.

## RESULTS

The overall response rate was 56% (71/127) from VA hospitals and 47% (415/881) from non-federal hospitals. Overall response rates between VA and non-federal hospitals did not differ significantly (*p*=0.08). The bed-size strata response rates for non-federal hospitals were 52% (78/150), 46% (208/451), and 46% (129/280) among hospitals with <50, 50–250, and >250 acute care beds, respectively. The bed-size distributions did not differ significantly between responding and non-responding hospitals for non-federal (*p*=0.42) or VA (*p*=0.71). Overall, 72% of surveys were completed on paper (44 VA and 306 non-federal), with the remainder being completed electronically.

Hospital characteristics are provided in Table 1. Non-federal hospitals had a larger average number of acute care (214 vs. 103 beds, *p*<.001) and intensive care unit beds (24 vs. 16, *p*<.001) compared to VA hospitals. VA hospitals were more frequently affiliated with a medical school (69% vs. 34%, *p*<.001). Non-federal hospitals more frequently reported very good to excellent support from leadership for infection control programs compared to their VA counterparts (64% vs. 44%, *p* <.001).

**Table 1:** Comparison of VA and Non-federal Hospital Characteristics

Question	VA (*n*=71)^a^	Non-federal (*n*=415)^a^	*p*-value
Number of adult acute care beds, mean (SD)	102.6 (103.7)	214.2 (218.2)	<.001
Number of intensive care unit beds, mean (SD)	15.7 (12.0)	24.0 (32.6)	<.001
Percent of rooms that are private, mean (SD)	64.3 (32.8)	80.3 (29.6)	<.001
Hospital affiliated with medical school, No. (%)	49 (69.0)	141 (34.2)	<.001
Hospital has hospital epidemiologist, No. (%)	39 (56.5)	161 (40.4)	.01
Hospital administrative leadership provides very good/excellent overall support of infection prevention program, No. (%)	31 (43.7)	266 (64.4)	<.001

^a^The *n* may vary for individual questions due to missing values

Table 2 provides hospital responses to various pandemic-related questions. Financial hardships stemming from the pandemic were considerably more common in non-federal hospitals (58% vs. 7%, *p*<.001) when compared to their VA counterparts. Furthermore, non-federal hospitals more frequently reported increased loss of staff due to resignations (76% vs. 53%, *p*=.001). The pandemic response plan was rated as very to extremely effective for most hospitals and did not differ significantly by hospital type (81% VA and 88% non-federal, *p*=.15). The Centers for Disease Control and Prevention (CDC) was relied upon most for information about COVID-19 in both organizations (91% VA vs. 83% non-federal, *p*=.08). A greater percentage of VA hospitals reported no shortages of sanitary and personal protective equipment supplies during the pandemic (17% vs. 9%, *p*=.03) and more frequently opened new units to care specifically for COVID patients (71% vs. 49%, *p*<.001). While fewer VA hospital infection preventionists reported that they were moderately or very confident that the COVID-19 vaccine will be safe (76% VA vs. 88% non-federal, *p*=.004), respondents at VA hospitals more frequently reported that their hospital’s vaccination plan has been very to extremely successful (77% vs. 62%, *p*=.01).

**Table 2:** Comparison of VA and Non-federal COVID-19 Response

Question	No. (%)	No. (%)	*p*-value
	VA (*n*=71)^a^	Non-federal (*n*=415)^a^	
Hospital’s pandemic response plan has been very/extremely effective in addressing COVID-19?	56 (81.2)	353 (87.6)	.15
Organization your facility relied on the most for information about COVID-19?^b^			
CDC	64 (91.4)	335 (83.1)	.08
State and/or local health department	12 (17.1)	106 (26.3)	.10
Local hospital/healthcare organization	8 (11.4)	23 (5.7)	.11
APIC	6 (8.6)	19 (4.7)	.24
WHO	4 (5.7)	8 (2.0)	.09
Other (e.g., CMS, IDSA)	1 (1.4)	15 (3.6)	.49
Hospital designated areas to care for COVID-19 patients separate from non-COVID patients.	64 (92.8)	350 (87.1)	.19
Hospital opened new units to care for COVID-19 patients	50 (71.4)	196 (48.6)	<.001
Hospital experienced staff shortages due to absences and/or illness during the COVID-19 pandemic?	60 (87.0)	355 (88.1)	.79
Hospital experienced an increased loss of staff in the midst of COVID-19	25 (53.2)	269 (75.8)	.001
Hospital experienced a shortage of supplies during COVID-19^c^			
N95 masks	45 (64.3)	320 (80.6)	.002
Disinfectant wipes	49 (70.0)	299 (75.3)	.35
Gowns	35 (50.0)	255 (64.2)	.02
Alcohol-based hand sanitizer	37 (52.9)	217 (54.7)	.78
Gloves	35 (50.0)	184 (46.3)	.57
Surgical masks	29 (41.4)	208 (52.4)	.09
Powered air-purifying respirators (PAPRs)	20 (28.6)	160 (40.3)	.06
Full face shields	19 (27.1)	141 (35.5)	.17
Eye shields/goggles	16 (22.9)	104 (26.2)	.56
Other (e.g., syringes, cleaning supplies)	1 (1.4)	41 (10.3)	.02
No supply shortages experienced	12 (17.1)	35 (8.8)	.03
Hospital experienced moderate/extreme financial hardship resulting from COVID-19?	2 (7.1)	171 (57.6)	<.001
Agree/strongly agree with “I feel safe carrying out my work role during the COVID-19 pandemic?”	63 (90.0)	373 (92.6)	.46
Moderately/very confident that COVID-19 vaccine will be safe and effective	53 (75.7)	354 (88.3)	.004
Hospital’s COVID-19 vaccination plan has been very/extremely effective in vaccinating staff	54 (77.1)	254 (62.0)	.01

^a^The *n* may vary for individual questions due to missing values

^b^Question initially designed to be select one option. Approximately 15% of respondents checked more than one response; therefore, data were analyzed as select all that apply

^c^Respondents were asked to select if any shortages were experienced and to select all that apply

## DISCUSSION

Our national survey of lead infection preventionists throughout the US revealed that while both VA and non-federal hospitals experienced shortages and challenges during the COVID-19 pandemic—an unanticipated worldwide system shock—the VA compared favorably overall and excelled in certain key domains.

Some findings were universal among all respondents. Both VA and non-federal hospitals had high rates of reliance on the CDC for COVID-19 information either as a primary source or in combination. Furthermore, the majority of hospitals received high marks by infection preventionists for their pandemic response plans, which is an encouraging indicator that evidence-based measures endorsed by the CDC and other expert bodies were implemented by hospital leadership. This is further reinforced by the concurrent high rates of creating designated COVID-19 care areas among all hospitals, a fundamental CDC recommendation.^19^ Unfortunately, the majority of respondents from both VA and non-federal hospitals also reported similarly high rates of staff outages due to absence or illness. This is understandable despite implementation of comprehensive infection control strategies within the hospital, as out-of-work community exposures have been repeatedly demonstrated to drive the risk of COVID infection in healthcare workers.^20–22^

VA hospitals excelled in a few key areas, most notably the reported lack of moderate/extreme financial hardship. This is readily explained by the difference in hospital funding mechanisms, with the VA receiving regular appropriations from the United States Congress while non-federal hospitals rely primarily on insurance and private party collections through a fee-for-service model. When elective and routine medical care were paused for months during the COVID-19 pandemic, revenue dropped drastically for most non-federal healthcare systems,^23^ whereas the VA’s medical care funding was increased by over $17 billion via the Coronavirus Aid, Relief, and Economic Security (CARES) Act.^24^ This likely explains the VA’s significantly lower rate of staff loss, as elimination of positions is one mechanism many non-federal hospitals employed to maintain financial solvency.^25^ Furthermore, a more robust workforce and financial resources may have enabled VA hospitals to more easily open new units to care for COVID-19 patients as compared to non-federal hospitals. This push for VA unit expansion was also influenced by the VA’s “Fourth Mission” activation, as sites increased capacity in preparation to care for non-Veterans where regional hospital resources were exhausted. In total, 27 VA hospitals opened inpatient beds to non-Veterans, caring for 697 non-Veteran patients. Furthermore, deployed staff completed 980 community nursing home and 122 non-VA hospital assignments, in addition to 3125 internal assignments.^5^ Of note, non-Veterans were generally satisfied with the care received at VA hospitals during the COVID pandemic.^6^

VA hospitals also performed favorably when queried about medical supply shortages, particularly for N95 masks, powered air-purifying respirators, gowns, and face shields—all key articles of personal protective equipment. In addition, the percentage of VA hospitals reporting “no experienced shortages” was more than double that of non-federal hospitals. The availability of needed resources is likely due to the VA’s extensive logistics network, which allows it to strategically cross-level resources internally at both the regional and individual facility level. Furthermore, the VA’s ability to use its vast purchasing power to create large-scale contracts directly with manufacturers and vendors streamlines the supply acquisition process for VA facilities. Resources obtained through this network were ultimately shared within the community via the Fourth Mission activation, with over 1.1 million pieces of personal protective equipment and medical supplies being sent to state and local facilities across the country.^5^

There are a variety of differences between the VA and many of its non-federal counterparts which may have allowed the VA to handle this unanticipated system shock more favorably. As described in a 2019 systematic review by Vaughn and colleagues,^1^ five interrelated domains exist that prevent healthcare systems from successfully improving quality, of which system shock is one. The others include poor organizational culture, inadequate infrastructure, lack of cohesive mission, and dysfunctional external relationships. The VA has inherent advantages in many of these domains. For example, its organizational design extends from beyond the facility to include both a robust regional and national reporting structure, allowing a layer of redundancy to mitigate vacancies in local leadership, along with a nimble mechanism to disseminate information and implement process change throughout the pandemic. In addition, the VA’s robust infrastructure network allowed for the rapid movement and cross leveling of supplies and equipment by logistics between VA facilities, while also leveraging underutilized facilities to readily expand capacity. Furthermore, having a large infrastructure creates less dependence on external relationships for routine hospital functions (e.g., inpatient transfers, external staff/service group contracts)—a common area of vulnerability when widespread scarcities occur. It should be noted, however, that higher rates of academic affiliation among VA hospitals may have also contributed to the robustness of the VA response, as affiliated academic hospitals may have provided a ready avenue for the sharing of medical supplies or personnel. Lastly, the VA has a cohesive mission in its charge to care for our nation’s Veterans and their dependents. Activation of the Forth Mission cemented this even further, reinforcing priorities in a time when maintaining staff resiliency was paramount.

Our survey does have several limitations. First, the overall response rate of approximately 50% is lower than observed with previous iterations of this survey, which ranged from 59 to 72%.^15,16^ This decrement was not unexpected, however, given the pressing clinical and administrative needs in the midst of the ongoing COVID-19 pandemic and the integral role played by the survey respondents in the pandemic response. In addition, given the rapidly shifting national medical supply chain, the timing of survey receipt or completion may have influenced response, especially regarding hospital readiness and resource allocation. Second, we relied on respondent self-report from the lead infection preventionist at each hospital. While infection preventionists are key frontline participants in any healthcare system’s COVID-19 response, their knowledge of global hospital operations may vary or be incomplete depending on their level of involvement in collaboration with hospital leadership. Finally, while we sampled all VA hospitals and employed a robust stratified random sampling process among non-federal hospitals to achieve nationally representative samples, it is possible that responding hospitals differed from those not responding, potentially impacting generalizability. However, we found no statistically significant differences in bed-size distribution between responding and non-responding hospitals in the VA or non-federal samples.

## CONCLUSION

The COVID-19 pandemic has repeatedly stretched US healthcare systems up to and beyond their routine capacities, while also precipitating rolling supply and staffing shortages. Our national survey of lead infection preventionists, key personnel in directing and implementing hospital pandemic responses, reveals that there are inherent advantages the VA has had in mitigating this system shock, particularly related to financial and staffing stability, supply logistics, and capacity expansion. While the COVID-19 pandemic resolves, a proactive approach to improving overall hospital quality and support will be critical to ensuring US hospital preparedness for the future. Prior to another global pandemic or similar external system shock, there is an opportunity for non-federal hospitals, particularly those most vulnerable (e.g., small, single site, low-complexity, or rural facilities), to emulate these advantages the VA experienced through local or regional partnerships and strong alliances with larger healthcare systems, state governments, and hospital associations. Examples of possible measures to pursue include the following: the creation of agreements with local hospital partners to streamline load balancing between sites, proactive creation of regional niche roles based on hospital infrastructure (particularly surrounding complex care, bed expansion, and post-acute care), creation of regional supply caches and equitable distribution procedures, and joint testing/vaccination outreach efforts.

While expansion of the VA’s Fourth Mission to extend bed capacity, staff support, and medical supplies demonstrated the feasibility of a unified mechanism to rapidly extend federal assistance to beleaguered non-federal healthcare systems, the magnitude of services delivered was not remotely adequate to mitigate needs across the US. As experienced by countries outside the US, nationally sponsored healthcare systems can still be overwhelmed by prolonged disaster events despite their large scale and ability to rapidly shift resources.^26^ While VA has been successful in the past at providing emergency medical services during geographically focused natural disasters (e.g., hurricanes, wildfires), if it is to act effectively in this role in future large-scale pandemic responses, further resource investment and proactive collaboration with non-federal hospitals will be critical.

## Data Availability

Data are available by reasonable request of the corresponding author.
